# New Perspectives on the Use of Cannabis in the Treatment of Psychiatric Disorders

**DOI:** 10.3390/medicines5040107

**Published:** 2018-10-02

**Authors:** Maria Scherma, Paolo Masia, Matteo Deidda, Walter Fratta, Gianluigi Tanda, Paola Fadda

**Affiliations:** 1 Department of Biomedical Sciences, Division of Neuroscience and Clinical Pharmacology, University of Cagliari, 09042 Monserrato, Italy; mscherma@unica.it (M.S.); paolo.masia@unica.it (P.M.); matteo.deidda@unica.it (M.D.); wfratta@unica.it (W.F.); 2 Medication Development program, NIDA-IRP, NIH/DHHS, NIDA suite 3301, Baltimore, MD 21224, USA; gtanda@intra.nida.nih.gov; 3 Centre of Excellence “Neurobiology of Dependence”, University of Cagliari, 09042 Monserrato, Italy; 4 CNR Institute of Neuroscience – Cagliari, National Research Council, 09042 Monserrato, Italy; 5 National Institute of Neuroscience (INN), University of Cagliari, 09042 Monserrato, Italy

**Keywords:** phytocannabinoids, Δ9-tetrahydrocannabinol, cannabidiol, psychiatric disorders

## Abstract

Following the discovery of the endocannabinoid system and its potential as a therapeutic target for various pathological conditions, growing interest led researchers to investigate the role of cannabis and its derivatives for medical purposes. The compounds Δ9-tetrahydrocannabinol and cannabidiol are the most abundant phytocannabinoids found in cannabis extracts, as well as the most studied. The present review aims to provide an overview of the current evidence for their beneficial effects in treating psychiatric disorders, including schizophrenia, anxiety, and depression. Nevertheless, further investigations are required to clarify many pending issues, especially those relative to the assessment of benefits and risks when using cannabis for therapeutic purposes, thereby also helping national and federal jurisdictions to remain updated.

## 1. Introduction

**Figure 1:**
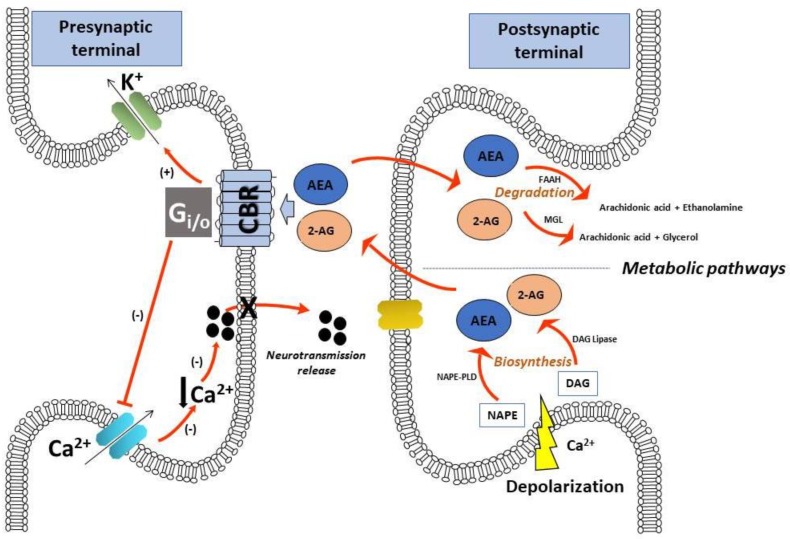
Schematic representation of the main elements of the endocannabinoid system at the synaptic level: endocannabinoids are produced upon demand after cellular depolarization from lipid precursors. AEA is derived mainly from NAPE by a specific phospholipase D; 2-AG is primarily formed from the hydrolysis of DAG. Once released from postsynaptic terminal, AEA and 2-AG activate CBRs in the presynaptic terminal, inhibiting neurotransmission release by activating presynaptic K^+^ channels and inhibiting Ca^2+^ channels. Endocannabinoid signaling is terminated by metabolic degradation by specific enzymes. AEA: anandamide; 2-AG: 2-arachidonoylglycerol; FAAH: fatty acid amide hydrolase; MGL: monoacylglycerol lipase; DAG: diacylglycerol; NAPE: *N*-acyl-phosphatidylethanolamine; NAPE-PLD: *N*-acyl-phosphatidylethanolamine phospholipase D; CBR: cannabinoid receptor; Gi/o: G protein; Ca^2+^: calcium; K^+^: potassium.

Evidence of the consumption of cannabis for therapeutic uses is prevalent throughout history. The first indication of use dates back to 2700 before Christ (BC), in the world’s oldest Chinese pharmacopeia, the Pen-ts’ao ching, which recommended cannabis for the treatment of constipation, malaria, gout, rheumatism, and painful menstruation, among others [1]. Around the year 1000 BC, the presence of cannabis was widespread throughout India, where its medical uses were numerous, owing to its analgesic, anti-inflammatory, anticonvulsant, appetite-stimulant, tranquilizing, and diuretic properties [2]. Subsequently, the plant gradually spread across the world. Cannabis use in Western medicine dates back to the first half of the 19th century, when Irish physician William Brooke O’Shaughnessy recommended it for a great variety of therapeutic purposes, including muscle spasms, menstrual cramps, and rheumatism, as well as convulsions of tetanus, rabies, and epilepsy [3]. Moreover, French psychiatrist Jean-Jacques Moreau de Tours experimented with the therapeutic use of cannabis in mental disorders, and described the plant as hypnotic, analgesic, and anticonvulsant [4]. In the second half of the 19th century, over 100 scientific articles were published regarding the therapeutic value of the plant, and cannabis extracts were listed for sedative and anticonvulsant effects in the British, and later United States (US), pharmacopeia [5]. However, the first few decades of the 20th century were characterized by decreased attention to the medical use of cannabis due to the social impact of increased drug consumption for recreational purposes, as well as to the fact that the effects were difficult to predict and standardize because of the variable composition of plant extracts. In the following years, cases of the medical use of cannabis completely subsided until the first decades of the 21st century, during which it returned to being considered for therapeutic purposes, despite its use being highly restricted [2]. The cannabis plant contains a total of 483 compounds, among which more than 120 are bioactive constituents, collectively known as phytocannabinoids [6]. Among them, the most important and studied are ∆9-tetrahydricannabinol (∆9-THC) and cannabidiol (CBD), which also represent the major constituents found in the plant. CBD was first isolated in 1940, but it was not until 1963 that its structure was clarified [7,8]. On the other hand, Δ9-THC was isolated in 1964, and was soon thereafter synthesized and found to be the primary psychoactive constituent of cannabis [9,10]. Primarily, the pharmacological effects of cannabis were attributed to the ability of ∆9-THC to alter specific membrane properties due to its high lipophilicity [11]. Nevertheless, evidence of it being able to inhibit the formation of adenylate cyclase [12] instigated researchers to hypothesize on the existence of a specific receptor for this compound. This receptor, named CB1 (CB1R), was cloned for the first time, in 1990, from a rat cerebral-cortex complementary DNA (cDNA) library [13], and it represents the most abundant G-protein-coupled receptor in the brain [14]. Later on, a second cannabinoid receptor, named CB2 (CB2R), derived from human promyelocytic leukemic cells (HL-60 cells), was cloned [15]. CB2R also belongs to the family of G-protein-coupled receptors, and it is mainly expressed in the immune system, while also being identified in some areas of the brain [16]. Recent studies suggest the existence of other cannabinoid receptors, including the orphan G-protein-coupled receptor, GPR55 [17]. Moreover, a growing body of evidence identified other receptors as cannabinoid targets, including the type 1 vanilloid receptor (TRPV1) and peroxisome proliferator-activated receptor [18]. The discovery of cannabinoid receptors prompted researchers to find endogenous ligands that activate them. The first endocannabinoid to be isolated, in 1992, was anandamide (AEA) [19]. Three years later, 2-arachidonoylglycerol (2-AG) became the second endocannabinoid to be identified [20]. In addition to AEA and 2-AG, several other compounds with endocannabinoid-like activity were isolated, including 2-arachidonylglyceryl ether (2-AGE, noladin), *O*-arachidonylethanolamine (virodhamine), and *N*-arachidonyldopamine (NADA) [21]. Soon after, the biochemical processes responsible for endocannabinoid synthesis and degradation were also identified [22]. AEA and 2-AG are synthesized upon demand in a Ca^2+^-dependent manner after cellular depolarization from lipid precursors, which are components of the cell membrane. AEA is derived mainly from the cleavage of *N*-arachidonoylphosphatidylethanolamine (NAPE), which is then specifically hydrolyzed by phospholipase D (NAPE-PLD); 2-AG is primarily formed from the hydrolysis of 1,2-diacylglycerol (DAG) by the phospholipase C/diacylglycerol lipase pathway [23]. Once synthesized, AEA and 2-AG are immediately released by the postsynaptic terminal and activate cannabinoid receptors (CBRs) in the presynaptic membrane, inhibiting neurotransmission release by activating presynaptic K^+^ channels and inhibiting N- and P/Q-type Ca^2+^ channels [23]. Endocannabinoid signaling is terminated by rapid metabolic deactivation via specific enzymes after being taken up into the cell. AEA is primarily metabolized by the intracellular enzyme, fatty-acid amide hydrolase (FAAH), which breaks it down into free arachidonic acid. Ethanolamine, as well as the pharmacological blockade and genetic deletion of this enzyme, not only enhances AEA levels, but also amplifies its effects [24,25]. The compound 2-AG is metabolized into arachidonic acid and glycerol mainly by the enzyme monoacylglycerol lipase (MAGL) [23]. These metabolic pathways represent the key points in the regulation of endocannabinoid tissue levels. Altogether, these elements are part of the endocannabinoid system representing important lipid signaling, which was recently recognized as a modulator of a large variety of physiological processes, as well as of emotional responses and cognitive function [26] (Figure 1). Abnormalities in emotion and cognitive deficits are characteristic of several neuropsychiatric conditions; thus, a defect in endocannabinoid signaling might play a role in the pathophysiology of these disorders [27,28]. The present review addresses the current literature on the endocannabinoid system in the neurobiology of psychiatric disorders, specifically schizophrenia and mood disorders (anxiety and depression), and current evidence for the beneficial effects of phytocannabinoids in treating them.

## 2. Search Methods

A PubMed database search was performed using the combination of keywords: ‘‘Cannabis”, “phytocannabinoids”, “∆9-tetrahydrocannabinol”, “cannabidiol”, “endocannabinoid system”, ‘‘psychiatric disorders”, “psychosis”, “Schizophrenia”, “anxiety disorders”, and “depression”. The search was carried out considering both human and animal studies articles. Clinical trials in healthy subjects were also considered.

## 3. Therapeutic Potential of Cannabis Extracts on Psychiatric Disorders

### 3.1. Schizophrenia

Schizophrenia (SCZ) is a severe psychiatric disorder whose clinical features fall into three categories: positive symptoms that include hallucinations, delusions, disorganized speech, and catatonia; negative symptoms indicating disruption in the expression of emotions or difficulty in beginning and sustaining activities (e.g., depression, anhedonia, and blunted affection); and cognitive deficits in working and verbal memory, as well as in executive function and attention [29]. The more severe the negative symptoms and cognitive deficits are, the more marked the disability is [30]. SCZ affects around 0.5–1% of the population worldwide and tends to be chronic, with a substantial impact on quality of life [31]. Antipsychotic medications, which represent the main treatment for SCZ, reduce psychotic symptoms, but are not effective in all patients: 30–60% of them are refractory to all current treatments. Moreover, these drugs cause several adverse effects [32,33]. Thus, the discovery of new molecular targets for the development of novel medication is of critical importance. In the last few years, a cannabinoid hypothesis of SCZ was postulated, and the pharmacological modulation of the endocannabinoid system could be considered a potential therapeutic tool for SCZ treatment [34].

#### 3.1.1. Human Studies

Clinical studies showed altered endocannabinoid signaling in schizophrenic patients [35]. For instance, elevated levels of AEA were found in the blood and cerebrospinal fluid of schizophrenic patients that were normalized by antipsychotic treatment [36,37,38,39]. Furthermore, increased cerebrospinal fluid levels of AEA seem to be negatively correlated with psychotic symptoms, and this increase may represent a protective role against psychosis [38]. Altered CB1R densities in schizophrenic patients were also reported, even though results were not entirely consistent across studies. Accordingly, an increase [40,41,42], decrease [43,44], or even lack of alteration [45] in CB1R density was found in cortical regions that are thought to be linked to SCZ, such as the dorsolateral prefrontal cortex and the anterior cingulate cortex. Since CB1R changes were investigated using radioligand binding and quantitative autoradiography in postmortem tissue, the variation in techniques used could account for the discrepancies obtained in these studies. Moreover, inconsistent results may also be related to the use of antipsychotic treatment [46] or cannabis consumption [40]. On the other hand, in vivo brain-imaging techniques in schizophrenic patients reported elevated CB1R binding in the pons, nucleus accumbens, cingulate, and insular cortex [47,48,49]. Recent clinical evidence also supports a role for CB2R. Indeed, reduced expression of CB2R was found in peripheral blood mononuclear cells of untreated schizophrenic patients with first-episode psychosis [50], as well as in patients following treatment with olanzapine [37]. Finally, genetic polymorphisms in the *CB1R* gene (*CN1R*) were implicated in susceptibility to SCZ; however, other studies showed no association [51,52,53,54]. Moreover, a correlation between single-nucleotide polymorphisms in the *CB2R* gene (*CNR2*) and increased risk of SCZ were reported [51]. In addition to an alteration of the endocannabinoid system, epidemiological evidence suggests that cannabis use is linked to an increased risk of developing SCZ in genetically predisposed people, as well as to precipitate psychotic symptoms in schizophrenic patients [55]. Early onset of use greatly increases the risk: meta-analysis studies indicated that adolescent cannabis use may account for 8–14% of SCZ cases [55,56]. On the other hand, the high rate of cannabis use in several schizophrenic patients was interpreted as an attempt to self-medicate negative symptoms or to overcome the feeling of depression and anxiety associated with these symptoms [57]. As mentioned earlier, ∆9-THC is the main psychoactive component of cannabis, and it is the active ingredient responsible for psychotic outcomes. Indeed, acute ∆9-THC administration elicits both positive and negative symptoms, as well as cognitive deficits in healthy individuals, while also inducing a transient exacerbation in psychotic symptoms and cognitive deficits in schizophrenic patients [58,59]. Neuroimaging studies showed that ∆9-THC-induced psychotic symptoms are associated with the altered activity of several brain areas affected by SCZ, including the prefrontal cortex (PFC), anterior cingulate cortex, amygdala, and ventral striatum [60,61]. By contrast, despite the lack of evidence supporting ∆9-THC-based medical treatments for SCZ, a recent study reported that three out of six treatment-resistant patients improved their schizophrenic symptoms following treatment with the synthetic form of ∆9-THC (dronabinol) [62]. Unlike Δ9-THC, CBD represents the most abundant non-psychoactive component of cannabis; it shows low CBR binding and partly antagonizes the actions of Δ9-THC and other synthetic CB1R agonists like WIN-55212 and CP-55940 [63]. Moreover, CBD inhibits AEA hydrolysis, stimulates vanilloid receptors like TRPV1, activates 5-HT1A receptors, and also exerts partial agonist activity on dopamine D2 receptors [64,65]. Recently, CBD received growing attention for its antipsychotic properties; thus, it could be considered a promising new agent in the treatment of SCZ [66,67]. Firstly, in healthy subjects, CBD blocked the psychotic symptoms induced by Δ9-THC [68]. Moreover, randomized clinical trials (RCTs) evaluating the clinical efficacy of CBD in psychosis found that it was able to improve both positive and negative symptoms in schizophrenic patients [32,69,70,71]. Furthermore, the clinical efficacy of CBD was comparable with that of amisulpride, but with fewer side effects [72]. Although the mechanism through which CBD exerts its antipsychotic effects is still to be clarified, the majority of studies have focused on its ability to directly inhibit the reuptake of AEA. In agreement, in the RCT carried out by Leweke et al. [72], CBD increased AEA serum levels, and this increase was associated with a reduction in psychotic symptoms. However, other molecular targets were proposed. The partial agonist activity on dopamine D2 receptors might, at least in part, account for CBD’s antipsychotic effects, and other authors suggested that it might act via 5-HT1A or TRPV1 receptors [66]. It is important to note that CBD is characterized by a more favorable safety profile, with only a few minor side effects, such as tiredness, diarrhea, and changes in appetite/weight [66].

#### 3.1.2. Animal Studies

Consistent with clinical observations, preclinical data also showed an involvement of the endocannabinoid system in the pathophysiology of SCZ. For example, the pharmacological blockade of AEA degradation improved negative symptoms in both amphetamine- and phencyclidine-treated rats [73,74]. CB1R alterations in various animal models of schizophrenia-like disorders were also found [28]. Using the model of social isolation rearing, a decrease in CB1R expression was found in the caudate putamen and amygdala of phencyclidine-treated rats, whereas an increase was observed in the ventral tegmental area and amygdala [75,76]. Moreover, the administration of a CB2R antagonist exacerbated the MK-801- or methamphetamine-induced disturbance of prepulse inhibition involved in acoustic startle and hyperactivity in mice [77]. Preclinical studies also support the hypothesis that CBD might have antipsychotic properties. For example, CBD attenuated the amphetamine-disruptive effects on prepulse inhibition, as well as the hyperlocomotion induced by psychotomimetic drugs [78,79].

### 3.2. Anxiety Disorders

Anxiety disorders are chronic, disabling conditions, including several syndromes such as generalized anxiety disorder (GAD), panic disorder (PD), social anxiety disorder (SAD), obsessive–compulsive disorder (OCD), and post-traumatic stress disorder (PTSD) [80]. These disorders represent the most prevalent mental illnesses in the world, with high societal costs [81]. Antidepressants and benzodiazepines are the main pharmacological treatments; however, 40–60% of patients do not attain total relief from their impairing symptoms [82]. Thus, there is a strong need to develop alternative treatments. In this regard, there is increasing interest in the endocannabinoid system as an important component of the complex circuitry involved in the control of responses to anxiety [83]. It is well established that cannabis consumption affects anxiety-related behaviors in a dose-dependent manner, with low doses being anxiolytic and high doses inducing adverse events, including increased anxiety and panic [84,85]. Data from animal tests further support the evidence of the bidirectional effects observed in humans: low doses of cannabinoids produced anxiolytic-like effects, while high doses produced anxiogenic-like responses [85]. The mechanisms underlying the effects of endocannabinoids on anxiety-related responses occur through CB1R, which is highly expressed in key structures within the brain directly involved in the modulation of emotional behavior, such as the PFC, amygdala, and hippocampus [86]. The involvement of CB1R was clarified using CB1-knockout mice. Under basal conditions, untreated CB1-knockout mice exhibited an increase in basal levels of anxiety-like responses compared to wild-type animals [87,88,89]. Moreover, CB1 deletion consistently caused anxiety under aversive conditions [88,90]. Furthermore, the anxiolytic effects of benzodiazepines appear to be less efficacious in CB1-knockout mice [89]. In contrast, Marsicano et al. (2002) found no alterations in anxiety-related responses between mutant and wild-type animals [91]. Using conditional mutant mice, the importance of the location of CB1R on specific neuronal subtypes with regards to biphasic effects was also demonstrated. CB1R located in glutamatergic neurons accounted for the anxiolytic effects of low doses, while CB1R located in γ-aminobutyric acid (GABA)-ergic neurons seems to be involved in the anxiogenic consequences of higher doses [92,93]. Experimental studies in animals showed that exposure to stress, both acute and chronic, appears to impair endocannabinoid signaling, with reduced AEA content in several brain regions, such as the PFC, hippocampus, and amygdala [94]. FAAH genetic variation also impacts enzyme expression and activity, thereby increasing AEA levels and attenuating anxiety-like behaviors in both mice and humans [95]. In agreement, the pharmacological blockade of FAAH was shown to reduce anxiety in a variety of animal models, such as the elevated plus maze and light–dark box test, and these effects were enhanced under aversive stimuli [96,97,98]. Altogether, these data indicate that the endocannabinoid system is clearly implicated in the modulation of anxiety, and its dysregulation may result in anxiety disorders. Thus, pharmacological modulation which enhances its signal was suggested as a target for the treatment of these disorders, and proposed drugs include ∆9-THC and CBD, among others [99].

#### 3.2.1. Human Studies

It is well documented that patients with anxiety disorders, as well as subjects with high levels of anxiety, use cannabis to cope with their symptoms. For example, subjects with SAD seem to use cannabis as a form of “self-medication” [100]. Among returning veterans, the most frequently endorsed conditions for cannabis use were anxiety/stress and PTSD [101]. Moreover, several studies reported a strong correlation between PTSD symptom severity and the level of cannabis use [102]. When the effects of smoked cannabis were evaluated in PTSD patients, improvements in the Quality of Life Scale, pain scores, and the Clinician-Administered PTSD Scale (CAPS) were reported [103]. In agreement, Roitman et al. [104], in an open-label study, showed that, in patients with unremitted PTSD, treatment with orally absorbable ∆9-THC had beneficial effects on global symptom severity, sleep quality, frequency of nightmares, and PTSD hyperarousal symptoms. Importantly, a CB1R positron-emission tomography imaging study showed increased CB1R expression in the brains of individuals with PTSD compared to the control group [105]. The same authors also demonstrated that this increase was accompanied by a significant reduction in peripheral AEA concentration. Under other conditions, significant correlation between cannabis use and the prevalence of anxiety disorders was well documented [106]. As mentioned above, the effects of cannabis on anxiety appear to be dose-dependent, with low doses producing an anxiolytic effect and higher doses producing anxiogenic behavior. Accordingly, in healthy subjects, ∆9-THC was demonstrated to decrease and increase anxiety levels at low and higher doses, respectively [84]. Moreover, neuroimaging studies showed that, at specific doses, ∆9-THC could both increase and decrease negative emotional processing in healthy volunteers [107,108]. Unlike ∆9-THC, CBD showed anxiolytic effects in humans without inducing anxiogenic effects at high doses at baseline [109]. When co-administered with Δ9-THC, CBD was able to attenuate the anxiogenic effect of high doses of Δ9-THC [68,110]. Moreover, CBD was able to reduce post-stress anxiety in healthy subjects submitted to simulated public speaking (SPS), and this effect was comparable with that of isapirone, a selective 5-HT1A-receptor partial agonist [111]. Using the same procedure, CBD was also able to reduce an increase in anxiety induced by SPS on subjects with SAD [112]. Neuroimaging studies showed that the anxiolytic effect of CBD was related to functional changes in brain areas involved in the control of emotional processes. In healthy volunteers, as well as in patients with SAD, CBD induced a significant decrease in anxiety, as determined by single-photon emission computed tomography (SPECT), acting predominantly in limbic and paralimbic cortical areas (amygdala and the hippocampus, as well as the hypothalamus, the left posterior cingulate gyrus, and the left parahippocampal gyrus), which are usually implicated in the pathophysiology of anxiety [113,114]. Finally, CBD enhanced the extinction of fear memories in healthy subjects, acting at the amygdala and the anterior and posterior cingulate cortex [107].

#### 3.2.2. Animal Studies

In agreement with the results obtained in clinical studies, preclinical evidence also showed the anxiolytic effects of CBD in several animal models of anxiety [109]. For example, in the elevated plus maze, CBD showed anxiolytic-like effects similar to diazepam in both rats and mice [115,116]. In the Vogel test, CBD also induced an anticonflict effect in rats, reducing the suppression of punished responses [117]. The use of these animal models also allowed the determination of the brain areas and the receptors involved in these effects. Indeed, anxiolytic effects were also found when CBD was microinjected into specific brain regions relevant to anxiety, including the central nucleus of the amygdala, the bed nucleus of the stria terminalis, and the dorsal periaqueductal gray [109]. Moreover, activation of the 5-HT1A receptor seems to mediate these effects, as they were attenuated by local treatment with the 5-HT1A-receptor antagonist, WAY100635 [118].

### 3.3. Depression

Depression is one of the most debilitating psychiatric disorders, affecting 20% of the population, characterized by sadness, emptiness, loss of interest or pleasure in everyday activities (anhedonia), impaired concentration or decision-making, psychomotor agitation or retardation, and insomnia or hypersomnia [119]. Antidepressants represent the first-line treatment prescribed for depression; however, not all patients achieve full remission, and many are unresponsive [120]. Consequently, depression tends to be chronic with high rates of recurrence and relapse [121]. In recent years, both clinical and preclinical evidence led to the hypothesis of a link between defects in the endocannabinoid system and depression [122].

#### 3.3.1. Human Studies

A reduction in serum content of the endocannabinoid 2-AG was found in females diagnosed with major depression, while serum AEA content was not significantly altered [123,124]. Moreover, the magnitude of 2-AG reduction strictly correlated with the duration of the depressive episode. On the other hand, the same authors also showed that exposure to an acute social stressor evoked a significant increase in serum 2-AG content in both females diagnosed with major depression and healthy matched controls [124]. In human postmortem studies, CB1R density and functionality, as well as CB1 messenger RNA (mRNA) levels, were found elevated in the dorsolateral prefrontal cortex of depressed suicides [125,126]. On the contrary, Eggan et al. [44] did not find alterations in CB1R mRNA and protein levels in the dorsal prefrontal cortex in subjects with major depression. Furthermore, the expression of CB1R in the anterior cingulate cortex was found to be reduced in depressed patients treated with serotonin selective reuptake inhibitors [45]. Genetic studies demonstrated a link between polymorphisms in the *CNR1* gene and increased vulnerability to developing a depressive episode following exposure to life stress [127], as well as increased risk of antidepressant resistance [128]. Both *CNR1* and *FAAH* gene polymorphisms might also contribute to susceptibility to bipolar disorder and major depression [129]. Finally, genetic variability in the *CNR1* gene seems to be involved in the etiology of major depression and in the clinical response to the selective serotonin reuptake inhibitor citalopram [130]. The evidence hereinbefore emphasizes that deficient endocannabinoid signaling may be implicated in the pathophysiology of depression; therefore, activation of the endocannabinoid system could represent a new pharmacological approach to treating patients. Anecdotal reports suggest that some individuals use cannabis to effectively treat depressive and manic symptoms [131,132,133]. For example, Gruber et al. [134] described five cases in which patients reported that cannabis relieved their depressive symptoms, and that they deliberately used it for this purpose. Moreover, a cross-sectional study showed that those who consume cannabis occasionally or even daily have lower levels of depressive symptoms than those who have never tried cannabis [134]. Babson et al. [135] reported that individuals with heightened depression symptoms had more severe problematic cannabis use because of the beneficial effects of cannabis on perceived sleep quality. Finally, in a recent systematic review, the authors identified seven cross-sectional studies in which there was clear evidence of an amelioration of depressed mood through the use of cannabis for medical purposes [136]. In bipolar disorder, it was found that some patients used cannabis to treat mania, depression, or both [131]. They also stated that it was more effective than conventional drugs, or helped relieve the side effects of those drugs. In agreement, an observational study showed that smoking cannabis acts to alleviate mood-related symptoms in at least a subset of bipolar patients [137]. Furthermore, two studies showed that cannabis use might be related to improved neurocognition in bipolar disorder [138,139]. On the other hand, a recent meta-analysis, including several longitudinal studies reporting on the association between cannabis and depression, concluded that cannabis use, particularly heavy use, may be associated with an increased risk of developing a depressive disorder [140]. Moreover, women with depressive disorders who used cannabis regularly reported poorer mental-health-related quality of life [141]. Cannabis use might also worsen the occurrence of manic symptoms in those diagnosed with bipolar disorder, and might also be associated with an increased risk of onset of new manic symptoms [142].

#### 3.3.2. Animal Studies

Modifications of CB1R and other elements belonging to the endocannabinoid system were also reported in animal models of depression [143]. For example, exposure to chronic mild stress reduced 2-AG brain tissue concentration in the hippocampus [144], but increased it in the hypothalamus, midbrain [145], and thalamus [146], whereas AEA content decreased throughout the brain [145] or showed no changes [146]. Moreover, exposure to chronic mild stress increased CB1R density in the prefrontal cortex and decreased CB1R density in the hippocampus, hypothalamus, and ventral striatum [145]. Treatment with the antidepressants tranylcypromine and fluoxetine increased CB1R-binding density in the PFC and hippocampus, and tranylcypromine also reduced the tissue content of AEA in the PFC, hippocampus, and hypothalamus, while increasing 2-AG content in the PFC [147]. Nevertheless, the potential antidepressant action of its major constituents, Δ9-THC and CBD, was demonstrated in several animal models of behavioral despair, such as the forced-swimming test or the tail-suspension test [148,149]. Antidepressant effects were also evident when CBD was injected in a specific brain area with a key role in emotion, such as the PFC [150]. Genetic deletion of CB1R in mice led to the development of a phenotype characterized by depressive-like and anxiety-like behaviors, as well as by an anhedonic state and cognitive deficits [143].

## 4. Conclusions

Despite the growing knowledge base of neuropsychiatric disorder neurobiology, a high percentage of patients do not respond to first-line therapeutic interventions. Therefore, there is clearly a need for new, more effective treatments. The endocannabinoid system plays a key role in emotional responses and cognition function, and both clinical and preclinical studies suggest that dysregulation of its neuronal signaling may be involved in the pathophysiology of these disorders [27,28]. Thus, therapeutic strategies based on drugs that modulate endocannabinoid signaling may be useful in the treatment of neuropsychiatric disorders. Cannabis has been used for millennia for therapeutic purposes, and there are several anecdotal reports of its use as a form of self-medication for the alleviation of neuropsychiatric symptoms (e.g., anxiety, depression, and mania) [85,132]. On the other hand, epidemiological studies have consistently demonstrated that heavy cannabis use could be associated with the occurrence of psychiatric outcomes, especially in people at risk for psychosis or with mood disorders [59,140]. Thus, evidence supporting the use of cannabis for the treatment of neuropsychiatric disorders is equivocal, which is mainly due to the use of the whole plant. As we know, cannabis contains various phytocannabinoids, among which ∆9-THC and CBD are the major constituents. As already mentioned, ∆9-THC is the main psychoactive component of cannabis, and it is the active ingredient responsible for both psychotic or affective mental health outcomes [59,107]. In contrast, CBD represents the non-psychoactive component of the plant and has been found to have antipsychotic properties and to be anxiolytic [67,111,149]. The ratio of these two compounds in smoked cannabis varies, and accordingly, the effects on mental health also vary. For example, the use of cannabis containing high Δ9-THC and low CBD concentrations was associated with a higher risk of a first psychotic episode [151]. On the contrary, using cannabis with a high CBD content was associated with significantly lower degrees of psychotic symptoms [152]. In agreement, in healthy volunteers who smoked cannabis, it was demonstrated that individuals with hair traces of ∆9-THC only had higher levels of positive schizophrenia-like symptoms than those with hair traces of both ∆9-THC and CBD [153]. When RCTs investigated the effects of Δ9-THC and CBD separately in the management of psychiatric patients, preliminary results suggested that CBD may have potential efficacy in the treatment of psychotic and anxiety disorders [70,109]. Indeed, pretreatment of CBD significantly improves psychotic symptoms in schizophrenic patients and decreases anxiety in patients diagnosed with generalized SAD [71,72,112]. Also, CBD was well tolerated with only a few minor side effects [66]. Although the studies reviewed here provide clear evidence of the beneficial effects of CBD in the treatment of psychiatric disorders, RCTs with a small sample size and short duration limit its potential clinical utility. Moreover, in some cases, CBD was evaluated as an adjunct to traditional treatments [71]. Thus, further and larger RCTs will be necessary to confirm the efficacy and safety of CBD, as well as basic research to understand its potential mechanism of action.
